# Machine learning to predict final fire size at the time of ignition

**DOI:** 10.1071/wf19023

**Published:** 2019-09-17

**Authors:** Shane R. Coffield, Casey A. Graff, Yang Chen, Padhraic Smyth, Efi Foufoula-Georgiou, James T. Randerson

**Affiliations:** A Department of Earth System Science, Croul Hall, University of California, Irvine, CA 92697, USA.; B Department of Computer Science, Donald Bren Hall, University of California, Irvine, CA 92697, USA.; C Department of Civil and Environmental Engineering, Engineering Hall 5400, University of California, Irvine, CA 92697, USA.

**Keywords:** boreal forests, decision trees, fire management, random forests, vapour pressure deficit

## Abstract

Fires in boreal forests of Alaska are changing, threatening human health and ecosystems. Given expected increases in fire activity with climate warming, insight into the controls on fire size from the time of ignition is necessary. Such insight may be increasingly useful for fire management, especially in cases where many ignitions occur in a short time period. Here we investigated the controls and predictability of final fire size at the time of ignition. Using decision trees, we show that ignitions can be classified as leading to small, medium or large fires with 50.4 ± 5.2% accuracy. This was accomplished using two variables: vapour pressure deficit and the fraction of spruce cover near the ignition point. The model predicted that 40% of ignitions would lead to large fires, and those ultimately accounted for 75% of the total burned area. Other machine learning classification algorithms, including random forests and multi-layer perceptrons, were tested but did not outperform the simpler decision tree model. Applying the model to areas with intensive human management resulted in overprediction of large fires, as expected. This type of simple classification system could offer insight into optimal resource allocation, helping to maintain a historical fire regime and protect Alaskan ecosystems.

## Introduction

Globally, fire prediction has received increasing attention because of the health and climate impacts of fires and the fact that fire regimes have been changing. First, in terms of public health, fire aerosols contribute to over 300 000 premature deaths each year (Johnston et al. 2012). They are also associated with increased hospitalisations due to respiratory and cardiovascular illness (Johnston et al. 2007; Delfino et al. 2009; Liu et al. 2017; Cascio 2018). Second, in terms of climate, fires are responsible for both positive and negative feedbacks with the climate system. Fires contribute significantly to the global carbon cycle, emitting 2.2 Pg of carbon annually (van der Werf et al. 2017). Deposition of black carbon aerosols increases the absorbed solar energy, melting snow and ice at high latitudes (Flanner et al. 2007; Mouteva et al. 2015; Hao et al. 2016; Sand et al. 2016). As a competing feedback, direct changes to the local landscape may increase reflected radiation, resulting in surface cooling on timescales of years to decades (Randerson et al. 2006; Rogers et al. 2013; Liu et al. 2019). Third, fire regimes have been changing around the globe because of human management and climate change. On average, global fire activity has been declining, largely driven by land use in grassland, savanna, and tropical ecosystems (Andela et al. 2017). However, areas such as the northern boreal forests and Western USA have seen increased fire activity due to climate change and human-caused ignitions, with climate change threatening to exacerbate this trend in the future (Westerling et al. 2006; Liu et al. 2012; Liu and Wimberly 2016; Veraverbeke et al. 2017).

In the Alaskan boreal forests in particular, the impact of a changing climate has been pronounced. The region has experienced warmer summers, longer growing seasons and an increase in lightning. Because Alaska’s burn area has historically been lightning-limited, the increase in lightning has resulted in recent years having some of the most frequent ignitions and most burned area on record (Kasischke and Turetsky 2006; Kasischke et al. 2010; Veraverbeke et al. 2017). Kasischke et al. (2010) reported that for first decade of the 21st century, the boreal region of Alaska had an average annual burned area of 7670 km^2^, the largest in a 150-year record. With an area of 516 000 km^2^ for the boreal interior region, this corresponds to a fire return frequency of ~70 years – at least 30 years less than estimates of variability for the Holocene (Lynch et al. 2002). Increasing lightning and fire trends are expected to continue with future climate warming (Flannigan et al. 2005; Krawchuk et al. 2009; Romps et al. 2014; French et al. 2015; Young et al. 2017), with one study predicting a doubling of burned area by 2050 relative to 1991–2000 (Balshi et al. 2009). Such a changing fire regime threatens both the native peoples and ecosystems that are maladapted to modern fire frequencies. The huge fires and their impacts in recent years may warrant a rethinking of fire management; lands that have previously been limited-suppression zones could now require increased suppression effort to maintain contemporary burning levels and mitigate impacts to humans and vulnerable ecosystems.

Previous work has illuminated the environmental controls on fires and fire size in boreal forests. The controls are typically a combination of topography, vegetation, meteorology and human activity (Kasischke et al. 2002; Flannigan et al. 2005; DeWilde and Chapin 2006; Parisien et al. 2011a; Parisien et al. 2014; Sedano and Randerson 2014; Rogers et al. 2015). Topography has been shown to be relevant both in terms of slope and aspect. Steep slopes can help with rapid upward spread of fires. Aspect is relevant as it relates to tree species and the thickness of the surface duff layer; black spruce, for example, is more likely to dominate north-facing slopes. This species is more flammable than other conifers and has been shown to influence fire intensity and size (Kasischke et al. 2002; Rogers et al. 2015). The structure of the vegetation as fuel can also control the spatial structure of burn probability, with large areas of contiguous conifer forest more likely to burn (Parisien et al. 2011b). In terms of meteorology, the Canadian Forest Service has developed the Canadian Forest Fire Weather Index (FWI) System to rate fire danger, using weather parameters to represent moisture content in various fuel layers. The weather parameters include 1200 hours local standard time (LST) temperature, relative humidity, 24-h precipitation and 10-m wind speed (Van Wagner 1987). Although the FWI has been used as a predictor of fire size and emissions (Di Giuseppe et al. 2018), simpler variables such as vapour pressure deficit (VPD) and temperature can explain regional variability in fire activity, including fire size (Wiggins et al. 2016). VPD appears to be important in setting both ignitions and spread in boreal forests, with VPD anomalies explaining 45% of the variance in annual burned area (Sedano and Randerson 2014). This is likely because of the importance of VPD in determining the moisture content in dead vegetation (fuels) on short timescales, especially in fine fuels like standing dead grass and live mosses (Miller 2019). Extreme temperature has been found to be a major control on boreal fire size at many different spatial scales, whereas relationships between burned area and other variables, including wind, fuel type, fuel moisture, topography and road density, often vary considerably with spatial and temporal scale (Parisien et al. 2011a; Parisien et al. 2014). Road density is important because it regulates access to wildlands, shaping patterns of both ignition and suppression. Fires near human-populated areas are more likely to be suppressed and less likely to become large (DeWilde and Chapin 2006). The presence of flammable fine fuels near roads may also allow lightning strikes to cause more fires in those areas (Arienti et al. 2009).

Numerous types of fire prediction models exist, including both dynamical physical-based spread models and statistical models. Two examples of dynamical spread models that are commonly used by Alaskan fire management agencies are FARSITE (Finney 1998) and the Fire Spread Probability Simulator (FSPro) (Finney et al. 2011). FSPro is a geospatial probabilistic model for predicting fire growth over many days. FARSITE is a deterministic modelling system used on shorter timescales (1–5 days) with a single weather scenario. In terms of rapid prediction of fire growth from ignition with minimal training, a few tools exist, such as REDapp from the Canadian Interagency Forest Fire Centre (http://redapp.org/, accessed 20 August 2019) and the Fire Behaviour Prediction (FBP) Calculator (Forestry Canada Fire Danger Group 1992). Even these are quite complex in comparison to the models we present, relying on information about fuel composition and mechanistic equations for fire spread.

Several studies have investigated statistical models for fire spread and size, primarily based on meteorological indices (Preisler et al. 2009; Faivre et al. 2014, 2016; Butler et al. 2017; Di Giuseppe et al. 2018). One study used machine learning techniques, including random forests, to predict burned area in Portugal with instantaneous weather conditions at ignition (Cortez and Morais 2007). The models relied on ground-station data and were most accurate for predicting the area of small fires. Less research has focussed specifically on the conditional probability of a large fire given information available at the time of ignition. One study used logistic regression with a fire potential index to predict the probability of fires exceeding a specified threshold in the contiguous USA (Preisler et al. 2009). This work examined the fraction of fires that would become large, but did not attempt to identify which specific ignition events were most likely to become large. Also, classification techniques have rarely been evaluated in the context of fire prediction. One example is a study in Brazil that used machine learning classification to predict the risk of ignitions in different areas, but similarly did not attempt to identify which ignitions were most likely to become large (de Souza et al. 2015).

In this study, we present and evaluate a new framework for fire prediction: using machine learning classification to identify specific ignitions that are most likely to become large fires. This is accomplished with two simple driver variables, extracted near the time and place of each ignition point. The final model is a decision tree that can efficiently classify ignition events. This approach may be especially promising for predicting fires and their impacts in the boreal forests of Alaska, where many ignitions occur and suppression resources are limited. In preparing for a future with more and larger fires, this type of simple prediction system may prove useful for fire and ecosystem management.

## Methods

### Data

**Fig. 1.**
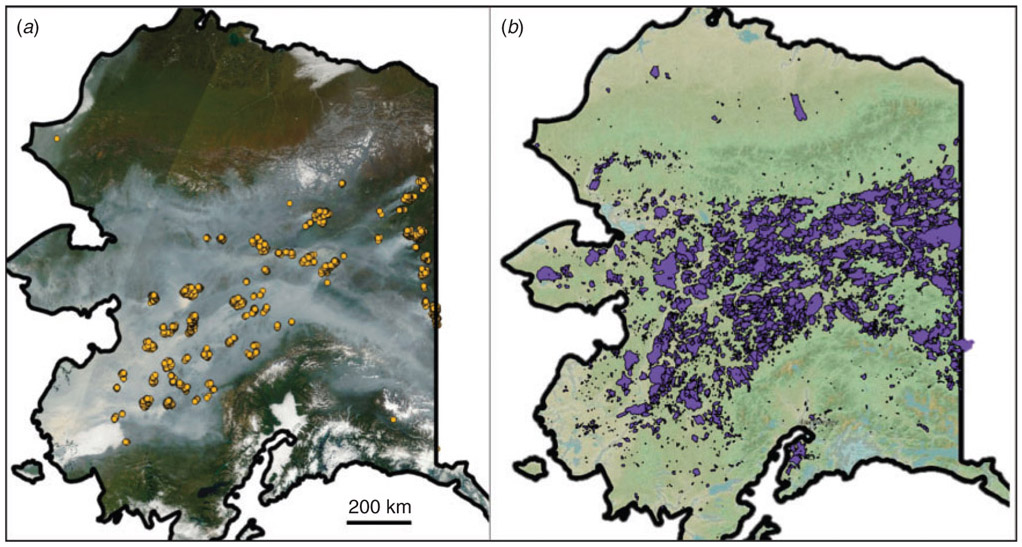
Study area of mainland Alaska, USA. In panel (*a*), Moderate Resolution Imaging Spectroradiometer (MODIS) active fire detections for 14 August 2005 are overlaid on a satellite optical image taken the same day (NASA EOSDIS). In panel (*b*), all fire perimeters from the Alaska Large Fire Database (ALFD) for 2001–2017 are overlaid on a background landscape map from QGIS Open Layers.

We chose as a study area the state of Alaska. The interior portion of Alaska is primarily a mixture of boreal forests and taiga which experience substantial burning (Wein and Maclean 1983; Kasischke et al. 2002). For example, in the large fire year of 2015, ~20 800 km^2^ of land burned. We chose a 17-year study period of 2001–2017, based on the availability of satellite and ground-based fire data as described below (Fig. 1). For each year, we considered the fire season of 1 May through 31 August, which contains fires accounting for 99.5% of the annual burned area according data obtained from the Alaska Large Fire Database (ALFD, http://fire.ak.blm.gov/incinfo/aklgfire.php, accessed 5 October 2018).

#### Fires

**Fig. 2.**
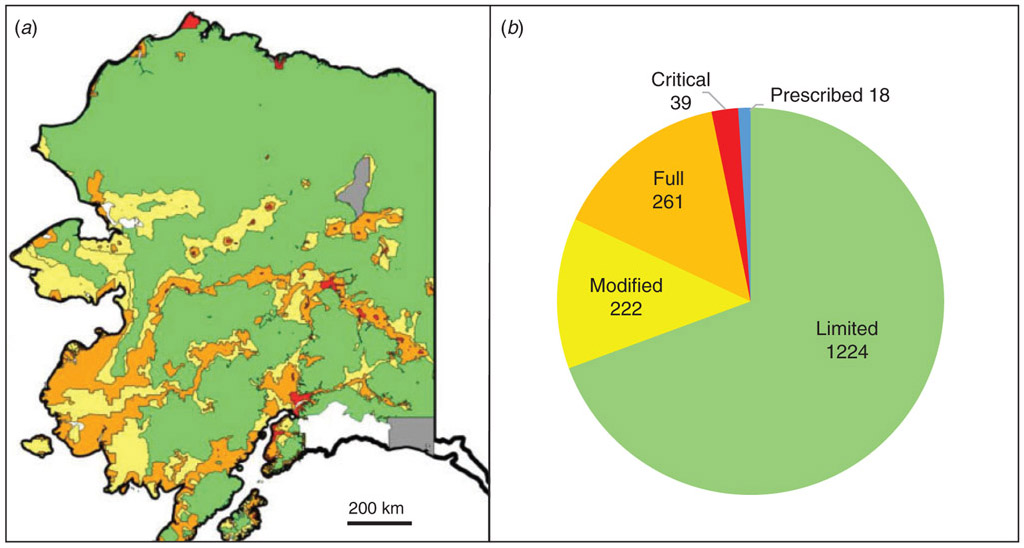
Prevalence of fires in different fire management zones. Panel (*a*) shows the fire management zones established by the Alaska Fire Service. Panel (*b*) shows the number of fires in the ALFD database that occurred in each zone during May–August of 2001–2017. In total, 1224 out of 1771 fires (69%) occurred in the limited management zone, where fires are more likely to be controlled by the natural environment and not suppression efforts. Out of the 1224 fires in the limited management zone, 1168 passed through an additional filter using satellite observations to corroborate the start date. This latter set was used in our model analysis.

We obtained historical fire perimeter data from the ALFD available through the Bureau of Land Management’s Alaska Interagency Coordination Center. The ALFD fire-history data compile information from satellite and ground-based records, reporting fire points, perimeters, start dates and management options back to 1939. For our time period, this gave a set of 1771 fires. The management options are determined by the Alaska Interagency Fire Management Plan (https://agdc.usgs.gov/data/projects/fhm/index.html, accessed 5 October 2018). They include ‘limited’, ‘modified’, ‘full’ and ‘critical’, in order of increasing priority for suppression resources (Fig. 2). Fires occurring in a modified, full, or critical zone are threatening to high-valued cultural or historical sites, high-valued natural resource areas, human property, or human life. Here, we selected only fires occurring in the ‘limited’ fire-management zone, which receives very minimal suppression, for two reasons. First, this set of fires had final fire perimeters that were more likely controlled by natural landscape and climate processes, and less by human intervention, making the modelling problem more tractable. Second, there is likely more flexibility in managing fires in this zone, making it an important potential target for efforts to maintain historical fire regions as a part of broader climate adaptation efforts. Considering fires only in this zone narrowed our dataset of fires from 1771 to 1224 fires.

We used active fire data from the Moderate Resolution Imaging Spectroradiometer (MODIS) to further filter the ALFD fire perimeter dataset. The MODIS Collection 6 Monthly Fire Location Product (MCD14ML) was obtained from the Department of Geographical Sciences at the University of Maryland (Giglio et al. 2016). Comparison of the ALFD and MODIS fire data revealed some spatial and temporal disagreement. In some cases, large fires in the ALFD had no corresponding fire detections from MODIS, and in other cases, the timing of fire events disagreed by multiple weeks. Since the start dates for some fires may be uncertain given the way multiple data sources are compiled in the ALFD, we compared start days with MODIS active fire detections to screen out potential outliers. We removed fires that were large (>4 km^2^) but had no associated MODIS detection within 10 km and 5 days, applying a reasonably wide temporal window for agreement as sometimes cloud or smoke cover can obscure fires for a few days. We did not filter out any fires in June 2001 when there was a gap in MODIS data. Our filtering further narrowed our dataset of fires from 1224 to 1168 fires.

#### Meteorology

We accessed daily meteorological data for 2-m air temperature, relative humidity, precipitation, 10-m wind speed and surface air pressure from the European Centre for Medium-Range Weather Forecasts (ECMWF) ERA5 reanalysis (Copernicus Climate Change Service 2017). The data are available at a 0.25° resolution. We used temperature and relative humidity to derive VPD. This deficit is the difference between the saturation vapour pressure and the actual vapour pressure; we calculated saturation vapour pressure using the Tetens equation (Tetens 1930). We also created a temperature anomaly variable by subtracting the mean temperature for each day over 2001–2017 from the observed temperature.

**Fig. 3.**
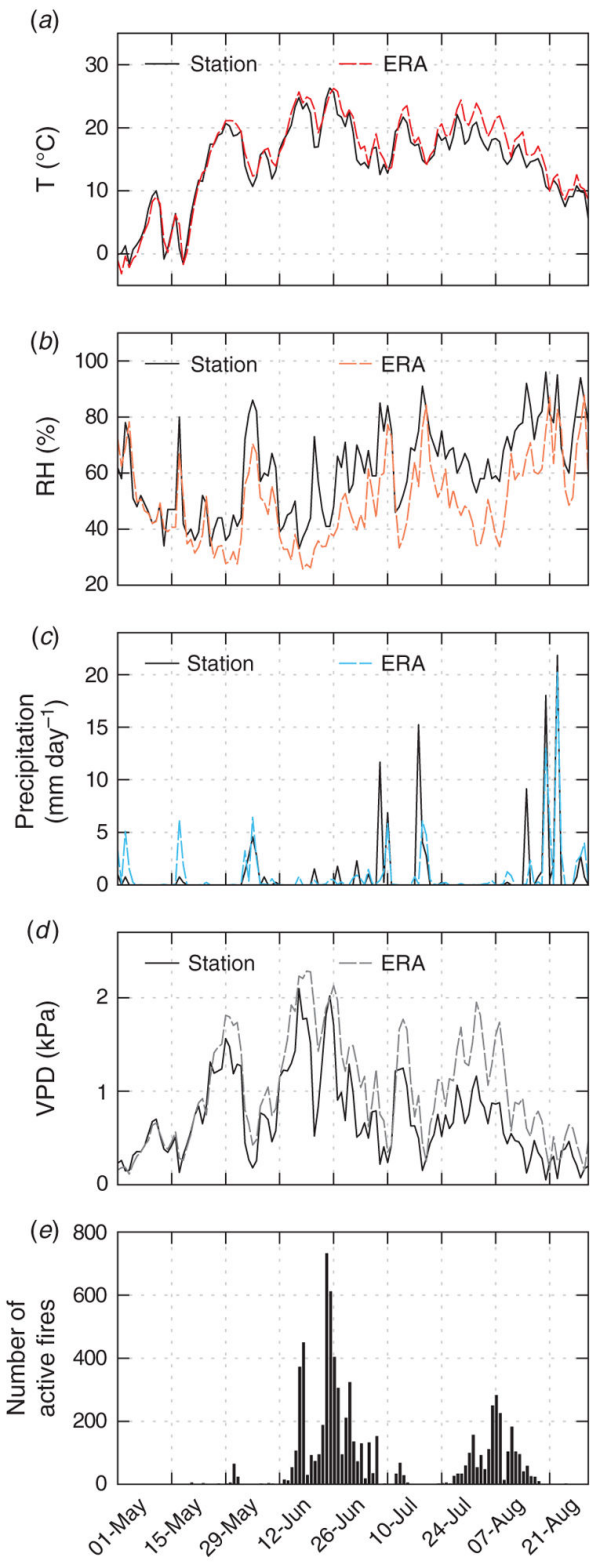
Time series of reanalysis weather data, ground station weather data, and fire activity for an example year, 2013. Panels (*a*–*d*) show the daily weather data from the European Center for Medium-Range Weather Forecasts (ECMWF) ERA5 reanalysis for the grid cell at Fairbanks along with *in situ* measurements from Fairbanks Airport station (from the Western Regional Climate Center). Despite the difference in spatial scale, total Moderate Resolution Imaging Spectroradiometer (MODIS) fire detections over interior Alaska (*e*) show a correspondence to weather, especially vapour pressure deficit (VPD).

As a preliminary validation of the ERA5 meteorology products, we plotted temperature, relative humidity, precipitation and VPD at Fairbanks through time for comparison against ground-truth weather data from the Western Regional Climate Center (https://raws.d.ri.edu, accessed 7 December 2018) (Fig. 3). The ERA5 global reanalysis appears to capture the local variability measured by the Fairbanks station. We also included a time series of the number of total fire detections in the interior region of Alaska (Fig. 3e). Total fire activity shows a strong correspondence to VPD in particular, despite the difference of spatial scales, given that Fairbanks is centrally located and the ERA5 data are spatially correlated across interior Alaska.

#### Vegetation

We included vegetation data from the LANDFIRE Existing Vegetation Type product, which is a Landsat-based classification available at a 30-m resolution for 2001, 2008, 2010, 2012 and 2014 (Rollins 2009). We created two vegetation classes, grouping together several abundant tree species known to influence fire behaviour: one class for any black or white spruce (evergreen) forest cover, and one class for any birch or aspen (deciduous) forest cover. For each fire, we considered these vegetation data at that location using the closest previous year that the data were available. We calculated the fraction of spruce forest cover and the fraction of birch–aspen forest cover for several different radii around each ALFD fire starting point.

#### Topography

Lastly, we included topographical data from the USA Geological Survey’s GTOPO30 global digital elevation model (DEM), available at a 30-arc second (~1-km) resolution (Gesch et al. 1999). Similar to the vegetation data, for each fire, we considered slope, elevation and aspect averaged for several different radii around each ALFD starting point.

### Model development and selection

We first developed and tested decision tree classifiers predicting final size class using data at the time and place of ignition. In contrast to many machine learning models, such as random forests or neural networks, decision trees are readily interpretable. Their interpretability and simplicity make them more transparent for applications in decision-support systems. They also allow us to draw more scientific insight into which variables, and in which combinations, are major controllers of final fire size.

We divided the population of 1168 fires from the ALFD into terciles and labelled them based on final burned area: ‘small’ corresponds to fires that burned less than 1.2 km^2^, ‘medium’ to fires between 1.2 and 19.8 km^2^, and ‘large’ to fires greater than 19.8 km^2^. It should also be noted that we briefly investigated using four or five fire size groups instead of three groups. We present only the three-size-group approach, given our fairly limited sample size with 10-fold cross-validation. Choosing three groups also makes the classification accuracy higher, which may be more useful for communicating with managers or the public.

In all cases, we used 10-fold cross-validation to develop and validate trees using the scikit-learn package in Python (Pedregosa et al. 2011). The scikit-learn decision tree classifier uses an optimised version of the Classification and Regression Trees (CART) algorithm, which relies on a standard Gini function to optimise leaf-node purity on the training set, and does not support pruning. More details on the algorithm is provided at https://scikit-learn.org/stable/modules/tree.html (accessed 20 August 2019). In cross-validation, we select models based on highest average accuracy on the test sets. The accuracy is defined as the number of correct classifications relative to the total number of classifications.

Because scikit-learn CART does not support pruning, for our analysis, we needed to specify the maximum size of the tree. In total, there were three dimensions to analyse in finding the optimal model: the tree shape, the timespan around ignitions to average weather data, and which variables to include.

As a starting point, we built decision tree classifiers based only on VPD averaged over a 5-day period from the day of ignition (*t* = 0) to 5 days in the future (*t* = 5). This window represents the idealised data that would be available in a standard weather forecast. We adjusted the size of the trees, allowing for up to 20 leaf nodes, and chose the tree shape with the highest accuracy in validation.

Next, we found the optimal timespan (around ignitions) over which to average weather data. We held the tree shape constant and varied the timespans of weather data, starting 10 days before ignition and ending 7 days after. Once the optimal timespan was selected, we analysed the information content in different input variables. We allowed the tree shape to change, and we report the highest accuracy of validation achieved (with error bars) using different combinations of weather variables.

In addition to the weather variables, we explored vegetation, topography and day-of-year (DOY) as model inputs. For the vegetation, we considered a spruce fraction and a birch-aspen fraction, averaged for a 4-km radius around each ignition point. We chose a 4-km radius because 4 km gave the largest correlation in a preliminary linear regression analysis between vegetation and burned area.

We tested four other machine learning classification algorithms in comparison to decision trees, all available through the same scikit-learn package in Python: random forests, k-nearest neighbours, gradient boosting and a multi-layer perceptron (MLP). For each, we manually searched over a range of relevant parameters and report model accuracy for the optimal parameter values.

### Model analysis

We chose a ‘best model’ based on highest validation accuracy and computed other statistics, including recall and precision, for large fires in particular. We developed and present a metric for the improvement in ‘weighted error’ over a null (random) classification model. This metric captures more information about misclassification. We defined accurate classification as error = 0, misclassification by 1 size class as error = 1, and misclassification by 2 size classes as error = 2. A random classification would have an average weighted error of (1/3) (0) + (1/3) (1) + (1/3) (2) = 1.

As another method of assessing model performance, we considered the cumulative burned area fraction accounted for when fires are ranked according to model prediction. Each fire in each test set was assigned a predicted probability of being in each size class. This allowed us to rank the fires in each test group by their predicted probability of being large. We show the mean and range of cumulative burned area fraction, derived from the 10 folds of data used in the cross-validation. We compare this modelled ranking to 10 simulated random rankings as well as the observed ranking based on observed fire size.

To assess whether the model could capture interannual variability in fire dynamics, we tested whether the best model was able to reproduce year-to-year differences in the fraction of large fires. In this case, we redeveloped models using each year as a hold-one-out fold for cross-validation (instead of 10 equalsized groups) and calculated the correlation between the observed and predicted fraction of large fires each year.

We also quantified the information content in the spatial *v*. temporal variability of the weather data. In one scenario, we used the climatological mean weather data for every grid cell as the input, regardless of when each ignition occurred. In a second scenario, we used the spatially averaged weather data for each day as the input, regardless of where on the landscape each ignition occurred. We report and compare the classification accuracies of these scenarios.

To explore the footprint of human fire management, we applied our best model, developed on fires in the ‘limited’ management zone, to fires occurring in other management zones where fires are more actively suppressed. By comparing fire sizes and quantifying the model’s overprediction of large fires in the other zones, we inferred how burned area was being modified by current fire management practices.

## Results

For our first set of models, we considered VPD averaged for each fire from the date of ignition through 5 days in the future. Allowing for trees with up to 20 leaf nodes, our ‘baseline’ best classification accuracy was 46.1 ± 6.7% using trees with 3 nodes. This represents the mean and standard deviation of accuracy across the 10 folds.

**Fig. 4.**
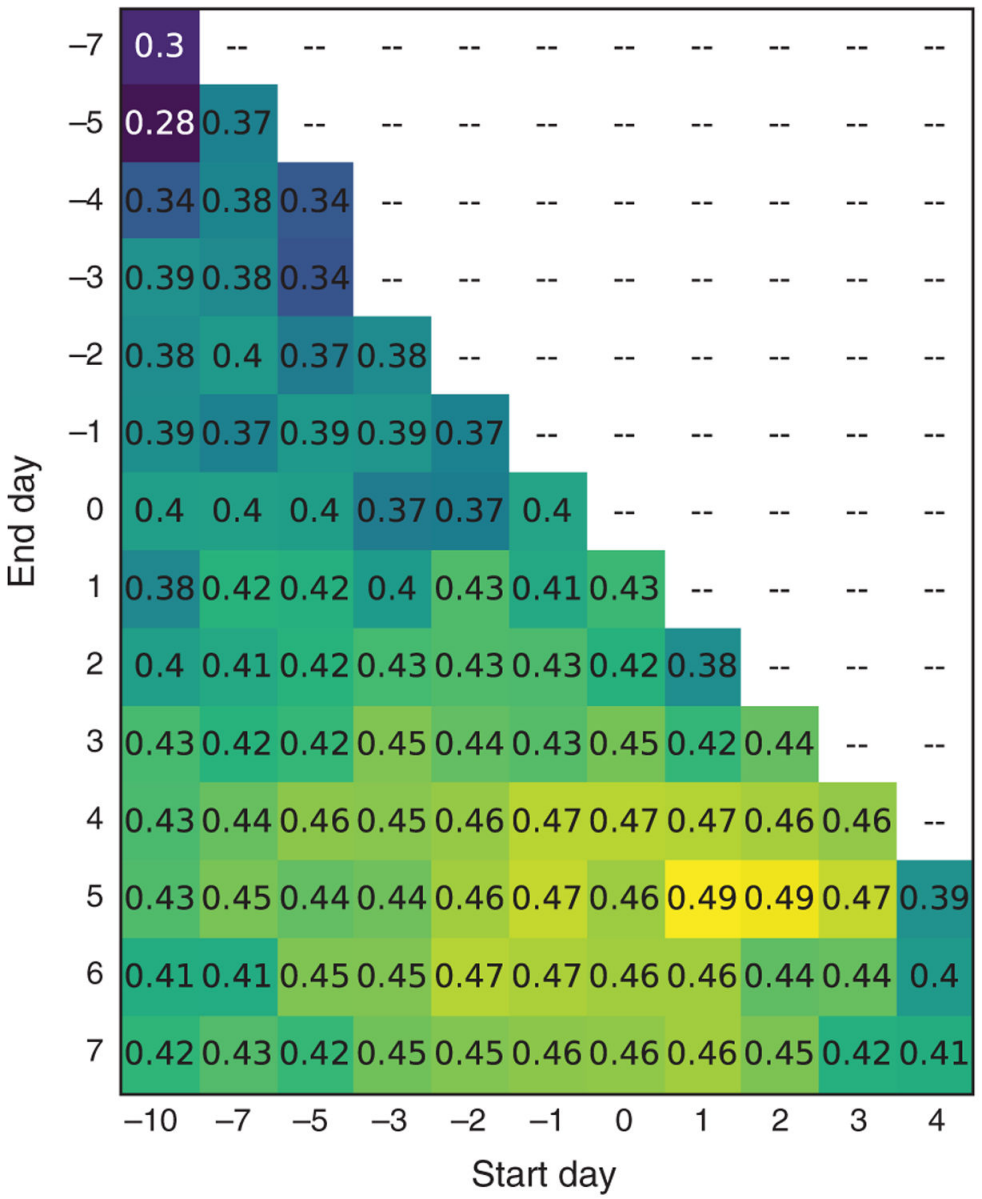
Classification accuracy with varying time window of weather data. Each cell shows the mean validation accuracy across the 10-fold cross-validation, using weather data averaged over different timespans. The timespans start up to 10 days before ignition (−10) and extend up through 7 days after ignition (+7). In all cases, classification models used only vapour pressure deficit (VPD) with 3 leaf nodes. From this analysis, the optimal time window for classification is from 1 to 5 days after ignition.

Next, specifying three-node trees, we averaged VPD data over different timespans. We found the optimal time window to be 1–5 days after the ignition, with an average accuracy of 49.2 ± 4.7% (Fig. 4). Going forward, we considered weather data over only this timespan for each fire.

Our analysis of weather variables is presented in Table 1. We found that VPD was the best predictor of final fire size at the time of ignition. Models including other weather variables did not outperform the VPD-only model. In addition to accuracy, we report *P*-values in Table 1, each representing a *t*-test comparing models with different variables against a random classification. All models except three (wind, surface pressure and temperature anomaly) significantly outperformed a random classification at a *P* = 0.05 level. It should also be noted that no models with combinations of variables significantly outperformed the models with only VPD or only relative humidity (RH).

Our analysis of other variables (day-of-year, vegetation and topography) is presented in Table 2. We tested all possible combinations of variables and report a select summary. Among the other variables, only two were statistically significant: day-of-year and spruce fraction. For the day-of-year variable, fires ignited in late June and early July were most likely to become large. However, including day-of-year did not improve the VPD model. For the spruce-fraction variable, fires with a low fraction of spruce forest around the ignition point were less likely to develop into the largest size class. This agrees with previous research highlighting the importance of black spruce trees in regulating fire intensity and severity in North America (Rogers et al. 2015). Including spruce fraction did improve the VPD model, although not significantly, with an accuracy of 50.4 ± 5.2%. For the remainder of this paper, we refer to this VPD plus spruce fraction model as our ‘best model’.

None of the more complex machine learning classifiers outperformed the simpler decision tree model (Table 3). For each classifier, we present the highest validation accuracy achieved, along with descriptions of the optimal parameters. Any parameters not specified were left at their default values.

**Fig. 5.**
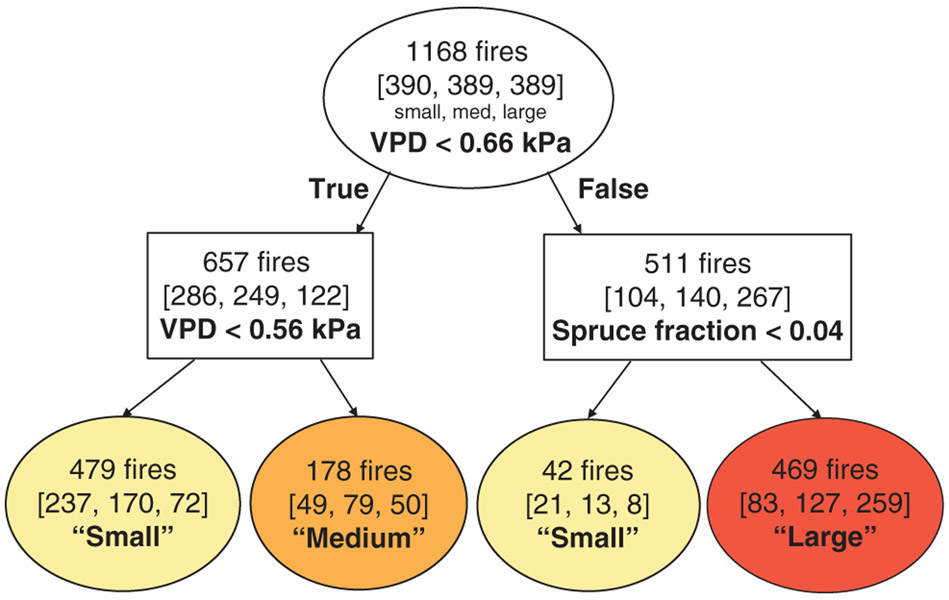
Example of a classification tree using vapour pressure deficit (VPD) averaged for 5 days after ignitions and the fraction of spruce cover in a 4-km radius. This representative tree results from training the entire dataset of 1168 fires. Thresholds for the splits are selected by the training algorithm, optimising leaf node purity. Colour coding with yellow, orange and red respectively indicates classification as small, medium or large fires. The numbers in brackets indicate the observed number of fires falling in each size class.

**Fig. 6.**
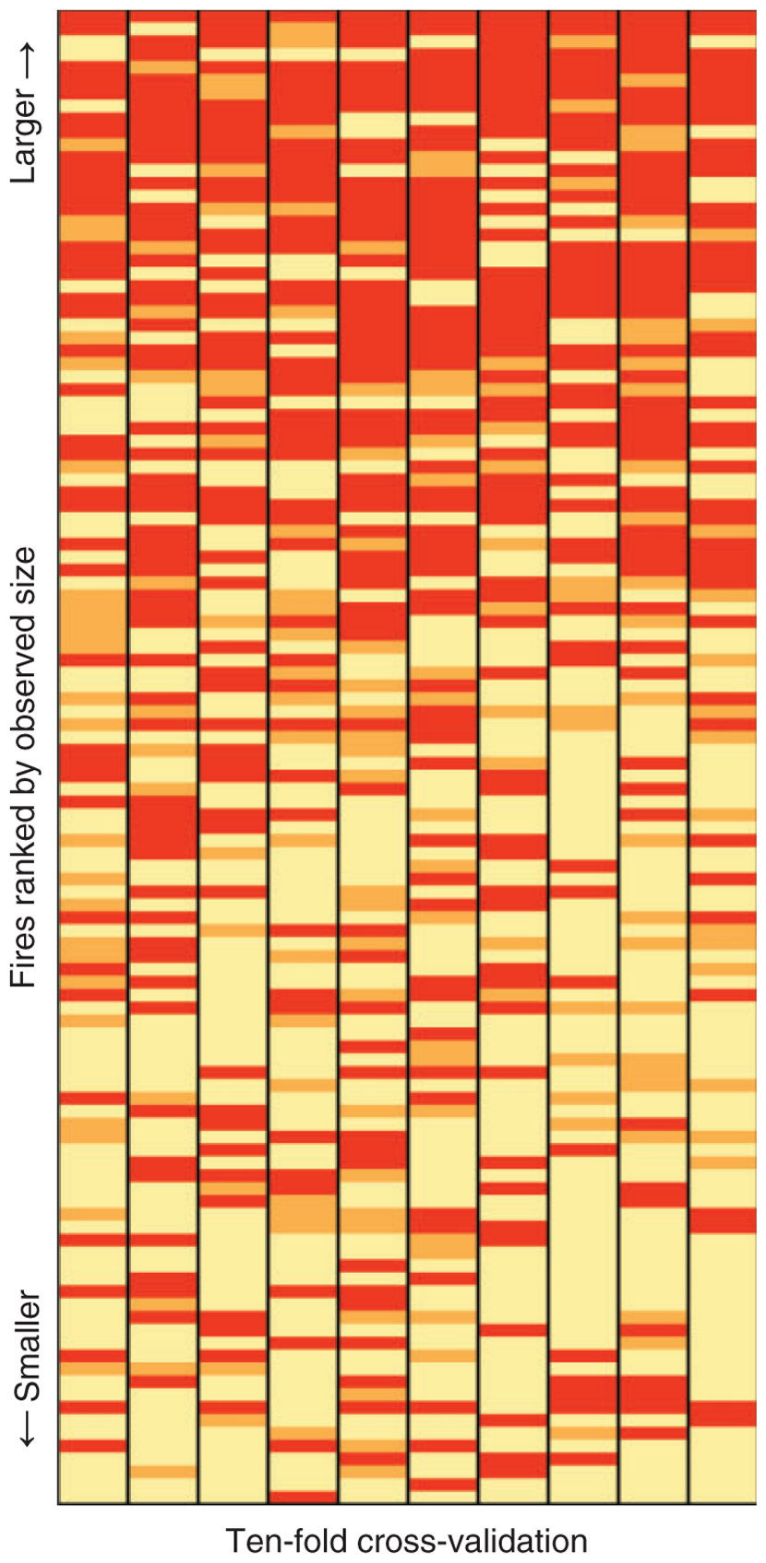
Performance of decision trees. Fires are separated into 10 columns representing the 10-fold cross-validation, with 116 samples in each fold. Fires are sorted vertically by the observed size from largest at top to smallest at bottom, and coloured based on model classification (red for large, orange for medium, and yellow for small).

For our best decision tree model, we present a representative tree (Fig. 5) and summary statistics (Table 4). In the tree, ignitions occurring during a period of low VPD were classified as small fires, and ignitions occurring during a period of moderate VPD were classified as medium fires. For ignitions occurring during a period of high VPD, most were classified as large fires. A subset of the high-VPD ignitions had a very low spruce fraction and were classified as small fires. Fig. 6 is a visualisation of the variation across the 10 folds. Our best model yields a weighted error of 0.637 ± 0.059, or an improvement (reduction) of 36.3 ± 5.9% over a random classification.

**Fig. 7.**
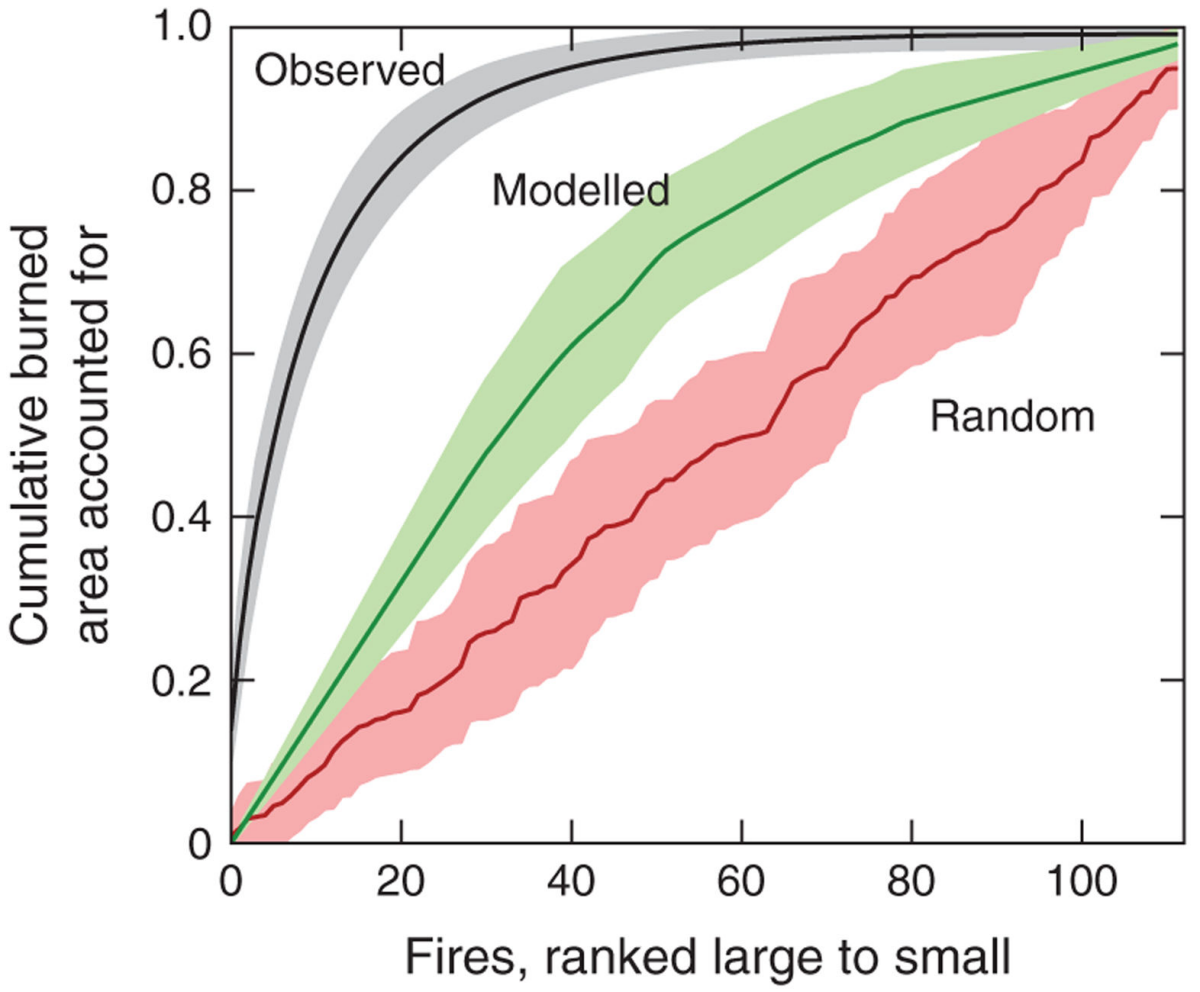
Cumulative burned area comparing observed, modelled, and random rankings of fires. Each line is a ranking of 116 fires on the *x*-axis. The errors about each line represent the variation from the 10-fold cross-validation. The ‘modelled’ line uses the vapour pressure deficit and spruce fraction model, ranking based on the predicted probability of each fire being large, as determined by the decision tree for that fold. The ‘random’ ranking is a numerical simulation in which all the fires are shuffled. In all three cases, the *y*-axis is the cumulative area that would be accounted for by each ranking system.

The model performed particularly accurately for the large fire class, with a recall of 65.2 ± 8.4% and a precision of 52.5 ± 11.8%. The model predicted that 40% of ignitions would become large fires. In reality, those 40% of ignitions became fires that accounted for 75% of the total burned area. In Fig. 7, we rank fires based on their modelled predicted probability of being large. This shows, for example, that half of the total burned area could be accounted for by the top 29% of fires identified by the model.

**Fig. 8.**
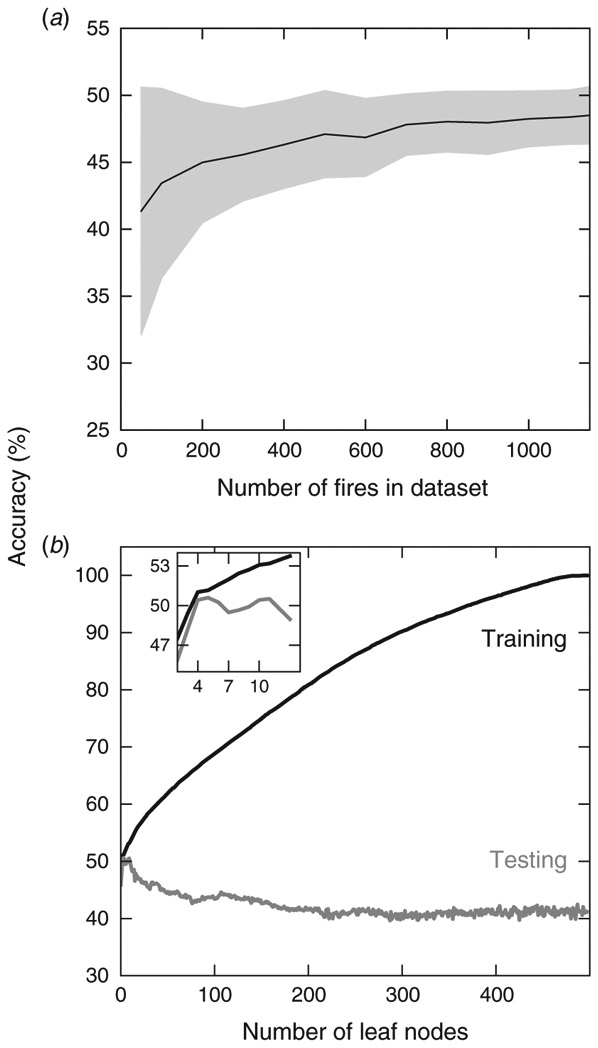
Learning curve and overfitting analysis. For (*a*), we randomly selected subsets of our fire dataset for model development and validation, and these subsets are ordered by size on the *x*-axis. The *y*-axis reflects the mean and standard deviation of model accuracy across 100 simulations. In each simulation, models were developed and validated using vapour pressure deficit and spruce fraction as inputs. The upper limit of accuracy with these parameters appears to be ~50%. The shape of the curve indicates that the accuracy of our model is not strongly constrained by data availability. For (*b*), we allowed to the number of leaf nodes to increase until each was pure. We chose the 4-leaf-node model as our ‘best model’.

Fig. 8 shows two more model assessments, investigating the role of (*a*) the number of fires in the dataset and (*b*) the number of leaf nodes in the decision trees. The number of fires in the dataset did not appear to be limiting model performance, as maximum accuracy approached 50% for as few as 200 fires. Also, overfitting did not appear to be limiting model performance, given that we selected our model based on optimal accuracy in the test group. A perfectly fit tree for the training dataset required 480 leaf nodes, but best performance for the test group was achieved with 11 or fewer nodes.

**Fig. 9.**
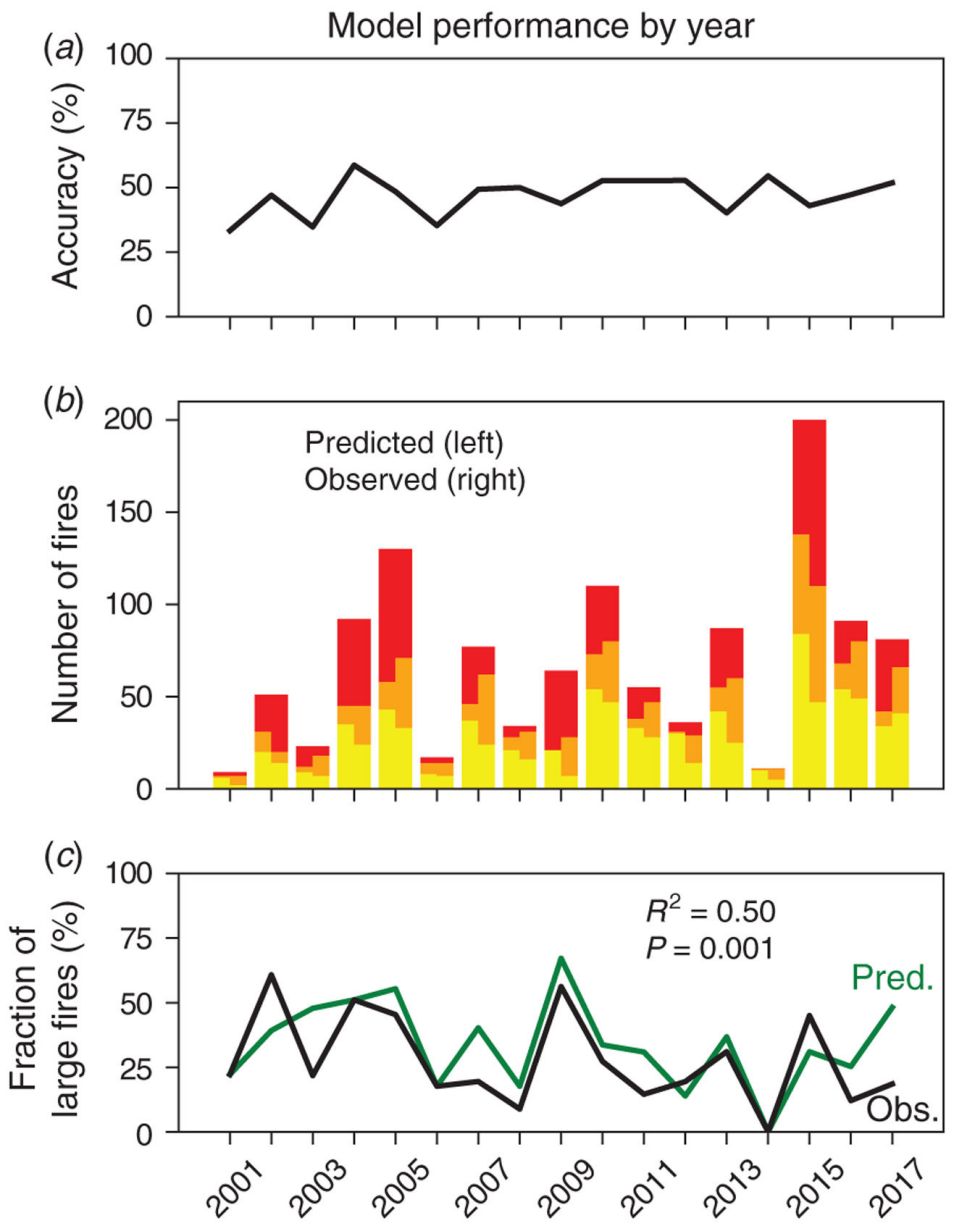
Model performance by year. We reran our best model using each year as a hold-one-out fold for cross-validation (instead of 10 equal-sized groups). Panel (*a*) shows model accuracy when tested on each year. Panel (*b*) shows the predicted (left) *v*. observed (right) fires falling into each size class each year (yellow for small, orange for medium, and red for large). Panel (*c*) shows the predicted (green) *v*. observed (black) fraction of large fires each year. The model generally captures the interannual variability of fires, predicting a larger proportion of large fires in 2004,2005 and 2009, but under predicting large fires in 2015.

On interannual timescales, the VPD plus spruce fraction decision tree model was able to capture year-to-year variations in the fraction of large fires (Fig. 9). The model correctly predicted the fraction of large fires increases during large fire years (Fig. 9c), as indicated by a significant correlation between predictions and observations during 2001–2017 (*r*^2^ = 0.50, *P* = 0.001).

We quantified the information in the spatial *v*. temporal variability of weather with the best model (Table 5). We found that these two components were comparable, with the ‘space-only’ model achieving an accuracy of 40% and the ‘time-only’ model achieving an accuracy of 41%. However, the two models varied significantly in which fire size classes were accurately captured; the ‘space-only’ model had higher recall for large fires, while the ‘time-only’ model had higher recall for small fires.

**Fig. 10.**
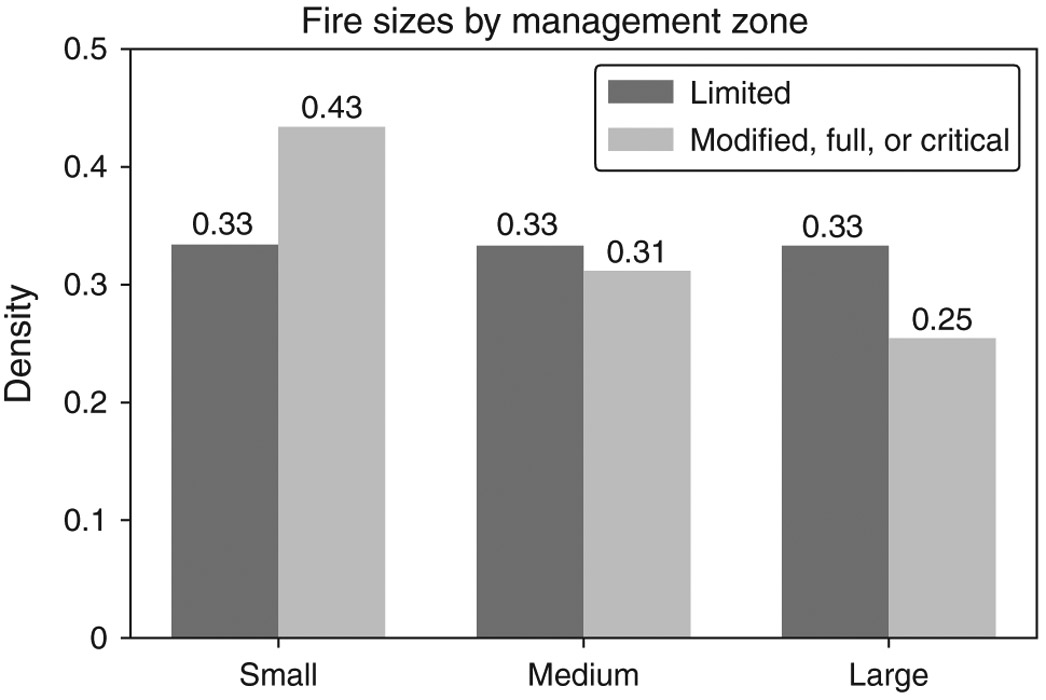
Fire sizes by management zone. The terciles of fires in the ‘limited’ management zone were used to define small (< 1.2 km^2^), medium (1.2–19.8 km^2^) andlarge (>19.8 km^2^). Fires in other management zones are less likely to become large, indicating the impact of suppression effort and human fragmentation of the landscape.

To quantify human impacts on Alaska’s fire regime, we considered fires in the other management zones that have a higher suppression priority. Specifically, we considered the combination of fires in the ‘modified’, ‘full’ and ‘critical’ management options. More fires in the high suppression zone were small (43%), and fewer became large (25%) (Fig. 10). Although there were 8% more ignitions per unit area in the high suppression zones, there was also 28% less annual burned area per unit area (Table 6). The increased fire frequency was likely explained by the higher density of roads, which allowed more ignitions by both humans and lightning, according to previous research (DeWilde and Chapin 2006; Arienti et al. 2009). Using Table 6, we estimated that the total human footprint on the fire regime in interior Alaska was to increase the frequency of fires by 3.4% but to decrease annual burned area by 7.5% during 2001–2017. The higher frequency of fires was more than offset by the increased suppression effort.

When applied to the other management zones (critical, full and modified), our model (using VPD and spruce fraction) overpredicted large fires. Accuracy decreased from 50.4 to 43.0%. Precision for large fires decreased from to 52.5 to 34.0%; however, recall for large fires stayed approximately the same, decreasing only slightly from 65.2 to 64.3% (Table 7). This drop in precision but not in recall aligned with intuition and supported the robustness of our model; the model did not predict large fires as precisely in these zones, as many of the fires that would have naturally become large were actively suppressed. However, the model still identified with the same success rate the fires that did become large, based on VPD and spruce fraction.

Moreover, we found that the overprediction of large fires in the more managed zones was disproportionate; for this set of ignitions, the model predicted 48.2% would become large (Table 7) rather than 40.2% (Table 4). Ignitions in the more managed zones were more often human-caused and occurred during periods of higher VPD, on average, than did those in the limited management zone (0.70 v. 0.66 kPa respectively). Using the mean fire size for each size class from the limited management zone, we found that our model predicted an average fire size of 1.8 times that which was observed for fires in the more managed zones. This suggests that suppression efforts decreased burned area in more managed zones by ~44%.

## Discussion

We present and evaluate a novel approach for fire prediction: decision tree classification with weather and vegetation cover data to predict final fire size at the time of ignition. We found that VPD alone, over the period of a standard weather forecast, could be used to classify ignitions into three groups with ~49% accuracy. VPD combined with one vegetation parameter, spruce fraction, improved accuracy to just over 50%. Further research could scale-up the complexity of the vegetation and topography variables to better capture the fuel structure and barriers to fire spread in the area around ignition.

Our findings suggest that weather, specifically VPD, early in a fire’s life can determine if a fire will be extinguished early or will be able to grow large. Further investigation is needed to compare the duration of fires in the small, medium and large classes in relation to the 5-day window used here. It may be that very dry conditions in the first few days allow the fires to grow large enough to persist through wet intervals, so that they can grow again during hot and dry intervals, as suggested by Sedano and Randerson (2014).

Our results are particularly promising for early identification of large fires. Accuracy was highest for the large fire class, with a recall of 65% and precision of 53%. The framework presented in Fig. 7 allows for a cost-benefit analysis of fire suppression. In theory, if it were possible to suppress fires at the instant of ignition, it may be possible to save 50% of the burned area by targeting only the top 29% of ignitions identified by our model. This type of information could offer substantial benefits for human health and preservation of vulnerable ecosystems as further climate warming increases burned area (Westerling et al. 2006; Liu et al. 2012; Liu and Wimberly 2016; Veraverbeke et al. 2017).

It is likely that weather forecasts would be a key limiting factor for model accuracy, as forecasts tend to degrade rapidly after a few days into the future. We did not investigate the degradation of model accuracy when using archived weather forecasts in place of reanalysis, primarily due to the cost of these ECMWF datasets. We speculate that the primary factor limiting accuracy to 50% is the incomplete characterisation of biology, fuels and barriers with our vegetation cover variables, which do not mechanistically account for fire spread. Information was also lost in our temporal averaging of weather and the inability of coarser-scale reanalysis products to capture very localised variations in precipitation. The number of fires in the dataset did not appear to be limiting the accuracy, based on a learning curve analysis (Fig. 8a).

With our approach focusing on information available at the time of ignition, we found that decision trees, a simple and readily interpretable method, performed similarly to other machine learning classifiers (namely, random forests, k-nearest neighbours, gradient boosting and multi-layer perceptrons). Incorrect application of any of these methods may yield overfitting, and so we provided an analysis of the training *v*. testing accuracy for our selected decision tree model (Fig. 8b). Although perfect training accuracy requires nearly 500 leaf nodes for a dataset of 1168 fires, testing accuracy is optimised for 11 or fewer leaf nodes. We did not include an analysis of more complex or deep learning methods (e.g. recurrent neural network), given our fairly small dataset and lack of indication that more complex models would outperform simpler models. However, future research in fire-size prediction should investigate more methodologies, especially at larger scales with more data and more complex input variables.

In our comparison of fire sizes and model results for different management zones, we also inferred the footprint of human suppression effort on burned area. As expected, our model overpredicts large fires in zones that are more actively managed. However, the model still had similar recall for the fires that did become large. Our model also allowed us to estimate the impacts of fire suppression, taking into account that human ignitions in these areas tended to occur during periods with hotter and drier weather.

Our models differed in structure and purpose from other fire size prediction methods and were not intended to compete with more complex models used for fire management. Rather, we view our analysis as a useful framework for investigating the major controls on fires using information available at the time of ignition. The insight gained may be useful in other regions beyond boreal forests of Alaska, where the early information could help inform management strategies in vulnerable ecosystems responding to strong trends in climate.

## Figures and Tables

**Table 1.** Information in different weather variables Decision trees are developed and validated including different combinations of variables. The mean and standard deviation of validation accuracy across the 10 folds are reported. Asterisks (*) indicate significantly higher accuracy than a random classification, and **bold** type indicates the highest-accuracy model. Tree shapes vary with up to 5 leaf nodes. RH, relative humidity; T, 2-m air surface temperature; Pr, total daily precipitation; VPD, vapour pressure deficit; W, wind speed; SP, surface pressure; T_anom_, temperature anomaly from climatology

Variables included	Accuracy of best model	*P*-value
	Random classification	
	33.3±4.4%	
	One-variable models	
RH	47.2±4.9%	<0.001*
T	39.4±6.4%	0.013*
Pr	45.7±5.0%	<0.001*
VPD	**49.2±4.7%**	<0.001*
W	29.6±9.0%	0.868
SP	31.6±9.7%	0.689
T_anom_	37.6±6.7%	0.055
	Two-variable models	
VPD, T	49.2±4.7%	<0.001*
VPD, Pr	48.8±5.5%	<0.001*
VPD, RH	47.8±3.8%	<0.001*
T, Pr	44.4±5.6%	<0.001*
T, RH	44.0±5.6%	<0.001*
Pr, RH	45.2±4.3%	<0.001*
	Three-variable models	
VPD, T, Pr	48.8±5.5%	<0.001*
VPD, T, RH	45.7±5.8%	<0.001*
VPD, Pr, RH	47.8±3.8%	<0.001*
T, Pr, RH	43.1±5.3%	<0.001*
	Four-variable model	
VPD, T, Pr, RH	45.5±5.9%	<0.001*

**Table 2.** Information in other variables (vegetation type, day of year, and topography) We tested all possible combinations of all variables and present a selected summary below. Asterisks (*) indicate significantly higher accuracy than a random classification, and **bold** type indicates the highest-accuracy model. ‘Spruce fraction’ is the proportion of black or white spruce cover in a 4-km radius around ignition; ‘Birch–aspen fraction’ is the proportion of birch or aspen cover in a 4-km radius around ignition. VPD, vapour pressure deficit

Variables included	Accuracy of best model	*P*-value
	Random classification	
None	33.3±4.4%	–
	One-variable models	
Spruce fraction	40.7±7.1%	0.007*
Birch-aspen fraction	29.4±4.8%	0.962
Day of year	39.1±7.3%	0.025*
Slope	36.2±7.1%	0.145
Aspect	26.4±7.6%	0.989
Elevation	34.7±6.0%	0.280
	Combination models	
Spruce, birch-aspen	40.4±7.2%	0.009*
VPD, spruce	**50.4±5.2%**	<0.001*
VPD, birch/aspen	49.2±4.7%	<0.001*

**Table 3.** Comparison of machine learning classification methods In addition to decision trees, we tested several machine learning classification methods. In each case, we manually searched over different combinations of relevant parameters and report the most accurate parameter settings for each model below. Performance of decision trees was effectively indistinguishable from that of random forests, and so we focus on the simpler model in this paper. VPD, vapour pressure deficit

Algorithm	Best accuracy	Variables included	Parameter description
Decision tree	50.4±5.2%	VPD, spruce	4 leaf nodes
Random forest	50.0±4.2%	VPD, spruce	6 leaf nodes, 32 trees
K-nearest neighbours	47.7±2.8%	VPD	60 neighbours
Gradient boosting	49.5±4.3%	VPD, spruce	50 estimators, 0.01 learning rate; depth 2
Multi-layer perceptron	48.0±3.6%	VPD, spruce	0.001 learning rate; single hidden layer with 50 units; 200 iterations; batch size 20

**Table 4.** Statistics for best model Models used vapour pressure deficit (VPD) and the fraction of spruce cover, with VPD averaged for the time interval of 1–5 days after the ignition event and spruce fraction averaged for a 4-km radius. We present the mean statistics across the 10-fold cross-validation. Recall is defined as the number of true positives divided by the sum of true positives and false negatives TP ÷ (TP + FN). It represents the proportion of observed large fires that were accurately identified by the model. Precision is defined as the number of true positives divided by the sum of true and false positives TP ÷ (TP + FP). It represents the proportion of fires the model predicted would be large that were observed large

Confusion matrix				
			Predicted	
		Small	Medium	Large
Observed	Small	22.2%	4.1%	7.1%
	Medium	16.2%	6.2%	11.0%
	Large	7.0%	4.2%	22.1%
Summary				
Accuracy				50.4±5.2%
Recall for large fires				65.2±8.4%
Precision for large fires				52.5±11.8%
Burned area accounted for by fires classified as large				74.9±12.6%
Improvement in weighted error over a null model				36.3±5.9%

**Table 5.** Information in spatial *v*. temporal variability of weather

Input data	Accuracy	Recall for small fires	Recall for large fires
Climatology for each cell (space only)	40.2±5.8%	33.9±24.8%	59.0±8.3%
Region-wide daily weather (time only)	41.1±7.0%	68.5±21.3%	40.4±22.5%
Daily weather for each cell	50.4±5.2%	65.7±8.3%	65.4±8.4%

**Table 6.** Summary of burned area and fire density across more managed zones Fires in the critical, full or modified management options of interior Alaska are more frequent but burn less area annually, per unit area. If the entire interior region followed the fire density and burn area density of the limited management zone, we estimate there would be (1.19 × 10^−4^ fires year^−1^ km^−2^) (633 581 km^2^) = 75.4 fires annually and (9.61 × 10^−3^ km^2^ year^−1^ km^−2^) (633 581 km^2^) = 6089 km^2^ burned area annually. By comparing against the observed values of 78.0 fires year^−1^ and 5631 km^2^ year^−1^, we infer that the human footprint is to increase the total number of fires only slightly, by 3.4%, but to decrease the total annual burned area by 7.5%

	Management option		
	Critical, full, or modified	Limited	All
Mean fire size (km^2^)	52.0	79.9	71.8
Burned area per year (km^2^ year^−1^)	1183	4449	5631
Fires per year	22.0	55.0	78.0
Area (km^2^)	170543	463038	633581
Burned area per year per area(km^2^ year^−1^ km^−2^)	6.94 × 10^−3^	9.61 × 10^−3^	8.89 × 10^−3^
Fires per year per area (*n* year^−1^ km^−2^)	1.29 × 10^−4^	1.19 × 10^−4^	1.23 × 10^−4^

**Table 7.** Statistics for best model applied to other management zones Models used vapour pressure deficit (VPD) and spruce fraction, with VPD averaged for the time interval of 1–5 days after the ignition event and spruce fraction averaged for a 4-km radius. This sample of 507 fires included management zones ‘critical’, ‘full’ and ‘modified’

Confusion matrix				
			Predicted	
		Small	Medium	Large
Observed	Small	22.2%	6.1%	15.0%
	Medium	9.9%	4.4%	16.8%
	Large	5.7%	3.4%	16.4%
Summary				
Accuracy				43.0%
Recall for large fires				64.3%
Precision for large fires				34.0%
Burned area accounted for by fires classified as large				70.6%
Improvement in weighted error over a null model				22.2%
